# Supplementary material for: Gene editing in the nematode parasite Nippostrongylus brasiliensis using extracellular vesicles to deliver active Cas9/guide RNA complexes
Source: Front Parasitol. 2023 Jan 26;2:1071738. doi: 10.3389/fpara.2023.1071738 (PMC11731642; doi:10.3389/fpara.2023.1071738)
Supplement: Supplementary Figure 1 — Alignment of cDNA and derived amino acid sequences for MN938457.1 (GenBank) and NBR_00001590 (Wormbase ParaSite). (A) cDNA sequence alignment. (B) Derived amino acid sequence alignment [file Image_1.pdf]

Supplementary Figure S1A. Alignment of mRNA for MN938457.1 (GenBank) and NBR_00001590 (Wormbase ParaSite).

```
MN938457.1        AAATTTGAAGTCATGTGGACAATCTTCCTCATAATTCCCGCCATCGTCGGGAATGTCGAT      60
NBR_00001590      ------------ATGTGGACAATCTTCCTCATAATTCCCGCCATCGTCGGGAATGTCGAT      48
                              ************************************************

MN938457.1        GCAGGTCTGAGTTGCAAGAACATGGAGGGCAAAGACGTGGACTGGTTCGCTGCCGTAAAG      120
NBR_00001590      GCAGGTCTGAGTTGCAAGAACATGGAGGGCAAAGACGTGGACTGGTTCGCTGCCGTGAAA      108
                  ******************************************************** **

MN938457.1        CTACCTTCCAATGTGGACGAGAGGAAGGGACGCACGTTTGCCTATTACGATTCCACACAA      180
NBR_00001590      TTACCTTCCAATGTGGACGAGAGGAAGGGACGCACGTTTGCCTATTACGATTCCACACAA      168
                   ***********************************************************

MN938457.1        ACTGGATGGAAGTTCAGCCCTCTACCGATTAACAGCACCGATTCCGCCATTGGTGCAACT      240
NBR_00001590      AATGGGTGGAAGTTCAGTCCTCTACCGATTAACAGCACCGATTCGGCTATTGGTGCAACT      228
                  * *** *********** ************************** ** ************

MN938457.1        GTTAAGCAACTCTACGACAGCGACAACTCCTATCACCTCAAAATCGCCTACAACGACGAC      300
NBR_00001590      GTTAAGCAACTCTACGACAGCGATAACTCCTATCACCTCAAAATTGCCTACAACGACGAC      288
                  *********************** ******************** ***************

MN938457.1        CATCCACATGGACACGAGGACAAGTCTTCAAGCGGTCGAGGCCACAGCAAGGGTGTCCTG      360
NBR_00001590      CATCCACATGGACACGAGGATAAGTCTTCAAGCGGTCGAGGCCACAGCAAGGGTGTCGTG      348
                  ******************** ************************************ **

MN938457.1        GTGTTCACCATTGAACGGGGATTCTGGCTGGTACACAGTGTGCCAAGATTCCCTGACCCC      420
NBR_00001590      GTGTTCACCATTGAACGGGGATTCTGGCTGGTACACAGTGTGCCAAGATTCCCTGACCCC      408
                  ************************************************************

MN938457.1        GAAAAATACGACTACCCCGAATCCGGCTCGAAATTCGCCCAGTCATTCATCTGTCTGACG      480
NBR_00001590      GAAAAATACGACTACCCCGAATCAGGCTCGAAATTCGCCCAGTCATTCATCTGCCTGACG      468
                  *********************** ***************************** ******

MN938457.1        TTGAGCTCTGATTTCCTTCCTGACATCAGCCAATATCTGCGCTATTCCCAGGTCACGCCG      540
NBR_00001590      TTGAGCTCTGATTTCCTGCCTGATATCAGCCAATATCTGCGCTATTCCCAGGTCACGCCG      528
                  ***************** ***** ************************************

MN938457.1        TTCGTCATGAATCTGCCCGAAAATCACAAATTACTGGCACCATACCTGGTCGACGTGCAG      600
NBR_00001590      TTCGTCATGAATCTGCCCGAAAATCACAAATTGCTGGCACCGTACCTGGTCGATGTGCAG      588
                  ******************************** ******** *********** ******

MN938457.1        GCAAAGAAGTCGCTAGGACGAGCTGATACCAAATTCACCTCGACTCATTCCTACCAGACA      660
NBR_00001590      GCAAAGAAATCGCTAGGACGAGCTGACACCAAGTTCACCTCCACTCATTCCTACCAGACA      648
                  ******** ***************** ***** ******** ******************

MN938457.1        ATGGGCGGAAAGCGATTCACGATTCTAGCGAAGCACAAGAAGTTCAACAACGACCTATGG      720
NBR_00001590      ATGGGCGGAAAGCGATTCACGATTCTAGCGAAGCATAAGAAGTTCGACAACGACCTATGG      708
                  *********************************** ********* **************

MN938457.1        CACGATTTCATCGCACTTTACTTCAAAACTCCCATGGCGGTGGAAACTTGGAGAAACGGT      780
NBR_00001590      CACGATTTCATCGCACTTTACTTCAAAACTCCCATGGCGGTGGAAACTTGGAGAAACGGT      768
                  ************************************************************

MN938457.1        GCTGCCAAAAACGTCGGAACCCAATGCGGCGTTGGATACAACGTCTACGACATTACCACA      840
NBR_00001590      GCTGCCAAAAACGTCGGCACACAATGTGGCGTTGGATATAATGTGTACGACATCACCCAA      828
                  ***************** ** ***** *********** ** ** ******** ***  *

MN938457.1        GTGAAAATTCTGGACAAAGTCTACAACAGCTCCAAGGACCACTCCAAATGGGGAGTGTCA      900
NBR_00001590      GTGAAGATTCTGGACAAAGTTTACAACAGCTCCAAAGATCACTCCAAATGGGGAGTGTCG      888
                  ***** ************** ************** ** ******************** 

MN938457.1        ATGGAGAAGAAAGAGCCCGTCGTTTGCATCGGAGATGTAAACCGACAGGAATCACAGTTC      960
NBR_00001590      ATGGAAGAGAGGGAGCCCCTAGTTTGTATCGGAGATGTAAACCGACAGGAATCGCAGTTT      948
                  *****  ***  ****** * ***** ************************** ***** 

MN938457.1        AAGCGCGGTGGTGGTGCTGTCTGCATGGAGGATGTCAAGCTGTGGAACACTTTCCACGAT      1020
NBR_00001590      AAACGCGGTGGTGGTGCTGTCTGCATGGAGGATGAGAAGCTGTGGAACACTTTCCACGAC      1008
                  ** *******************************  *********************** 

MN938457.1        TCGGTCAAGTCTTATTTGAACTGCGGAGAAGTCCAAGAAAGGAAGAGTAAAGACGAGGAC      1080
NBR_00001590      TCGGTCAAATCTTACCTGAACTGCGGAGAAGTCCAAGAAAGGAAAAATAAAGACGAGGAC      1068
                  ******** *****  **************************** * *************

MN938457.1        AACAAAACCGAGAGCAAACCAAAGAAGCCGAGCAAGAAGACAAACAAAACCGCC            1134
NBR_00001590      AACAAAACTGAAAGCAAACCAAAGAAGCCGAGCAAGAAGACAAACAAAACCGC-            1121
                  ******** ** *****************************************
```

Supplementary Figure S1B. Alignment of derived amino acid sequences for MN938457.1 (GenBank) and NBR_00001590 (Wormbase ParaSite).

```
MN938457.1        MWTIFLIIPAIVGNVDAGLSCKNMEGKDVDWFAAVKLPSNVDERKGRTFAYYDSTQTGWK     60
NBR_00001590      MWTIFLIIPAIVGNVDAGLSCKNMEGKDVDWFAAVKLPSNVDERKGRTFAYYDSTQNGWK     60
                  ********************************************************.***

MN938457.1        FSPLPINSTDSAIGATVKQLYDSDNSYHLKIAYNDDHPHGHEDKSSSGRGHSKGVLVFTI    120
NBR_00001590      FSPLPINSTDSAIGATVKQLYDSDNSYHLKIAYNDDHPHGHEDKSSSGRGHSKGVVVFTI    120
                  *******************************************************:****

MN938457.1        ERGFWLVHSVPRFPDPEKYDYPESGSKFAQSFICLTLSSDFLPDISQYLRYSQVTPFVMN    180
NBR_00001590      ERGFWLVHSVPRFPDPEKYDYPESGSKFAQSFICLTLSSDFLPDISQYLRYSQVTPFVMN    180
                  ************************************************************

MN938457.1        LPENHKLLAPYLVDVQAKKSLGRADTKFTSTHSYQTMGGKRFTILAKHKKFNNDLWHDFI    240
NBR_00001590      LPENHKLLAPYLVDVQAKKSLGRADTKFTSTHSYQTMGGKRFTILAKHKKFDNDLWHDFI    240
                  ***************************************************:********

MN938457.1        ALYFKTPMAVETWRNGAAKNVGTQCGVGYNVYDITTVKILDKVYNSSKDHSKWGVSMEKK    300
NBR_00001590      ALYFKTPMAVETWRNGAAKNVGTQCGVGYNVYDITQVKILDKVYNSSKDHSKWGVSMEER    300
                  *********************************** **********************::

MN938457.1        EPVVCIGDVNRQESQFKRGGGAVCMEDVKLWNTFHDSVKSYLNCGEVQERKSKDEDNKTE    360
NBR_00001590      EPLVCIGDVNRQESQFKRGGGAVCMEDEKLWNTFHDSVKSYLNCGEVQERKNKDEDNKTE    360
                  **:************************ ***********************.********

MN938457.1        SKPKKPSKKTNKTA                                                  374
NBR_00001590      SKPKKPSKKTNKT-                                                  373
                  *************
```
