# Supplementary material for: Gene editing in the nematode parasite Nippostrongylus brasiliensis using extracellular vesicles to deliver active Cas9/guide RNA complexes
Source: Front Parasitol. 2023 Jan 26;2:1071738. doi: 10.3389/fpara.2023.1071738 (PMC11731642; doi:10.3389/fpara.2023.1071738)
Supplement: Supplementary Figure 2 — Analysis of extracellular vesicle preparations. (A) Analysis of extracellular vesicle production by western blot. Extracellular vesicles (EV) or NanoMEDIC (Nano)-containing cell supernatants were concentrated using vivaspin columns (VIVA) or precipitation with Lenti-X concentrator (LX) as described in Materials and methods. Western blotting was performed following SDS-PAGE under reducing conditions to determine the presence of Cas9 and VSV-G, or under non-reducing conditions for the presence of CD63 and CD81. (B) Analysis by Nano-flow cytometry. NanoMEDIC preparations concentrated by ultrafiltration (VIVAspin) or precipitation (Lenti-X) were analysed for their nanoparticle content by nano-flow cytometry as described in Materials and methods. The table shows the concentration and proportion of particles with diameters larger than 100 nm. [file Image_2.pdf]

Supplementary Figure S2. Analysis of extracellular vesicle preparations.

A Analysis of extracellular vesicle production by western blot

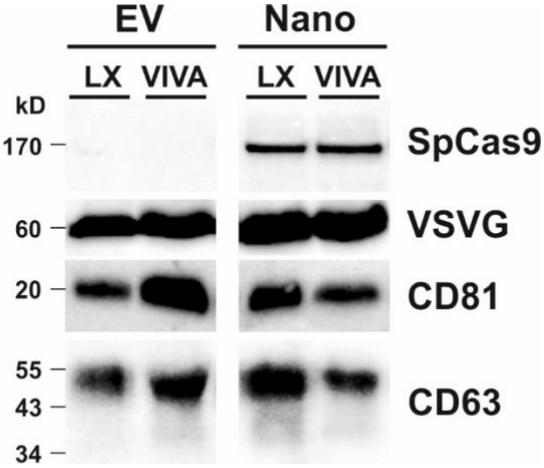

B Analysis by Nano-flow cytometry

	Lenti-X	*VIVAspin*
Sample concentration	1.88 x 10^{11}/ml	5.12 x 10^{10}/ml
Gated EVs (>100 nm)	7.7%	16.19 %
Gated EVs (>100 nm) concentration	1.45 x 10^{10}/ml	0.83 x 10^{10}/ml
