# Supplementary material for: Gene editing in the nematode parasite Nippostrongylus brasiliensis using extracellular vesicles to deliver active Cas9/guide RNA complexes
Source: Front Parasitol. 2023 Jan 26;2:1071738. doi: 10.3389/fpara.2023.1071738 (PMC11731642; doi:10.3389/fpara.2023.1071738)
Supplement: Supplementary Figure 3 — Guide RNA-encoding region synthesised for cloning into RGR plasmid and ssODN sequences for homology directed repair. Key: Red: guide RNA target sequence; Yellow: reverse complement of first 6 nucleotides of target; Blue: Hammerhead ribozyme; Green: Hepatitis Delta virus ribozyme; Lower case: restriction endonuclease sites. [file Image_3.pdf]

Supplementary Figure S3. Guide RNA-encoding region synthesised for cloning into RGR plasmid and ssODN sequences for homology directed repair.

Exon 3 guide 91

ggtaccGAATTCGCGGCCCACCACCTGATGAGTCCGTGAGGACGAAACGAGTAAGCTCGTCGTGGTGTTCACCATTGAACGGGGGTTTTAGAGCTATGCTGGAAACAGCATAGCAAGTTAAAATAAGGCTAGTCCGTTATCAACTTGAAAAAGTGGCACCGAGTCGGTGCTTTTTTTTTGGCCGGCATGGTCCCAGCCTCCTCGCTGGCGCCGGCTGGGCAACATGCTTCGGCATGGCGAATGGGACggccGCTCTAGA***gctagc***

Exon 3 guide 46

ggtaccGAATTCGCGGCCCTCGTGCTGATGAGTCCGTGAGGACGAAACGAGTAAGCTCGTCCACGAGGACAAGTCTTCAAGCGGGTTTTAGAGCTATGCTGGAAACAGCATAGCAAGTTAAAATAAGGCTAGTCCGTTATCAACTTGAAAAAGTGGCACCGAGTCGGTGCTTTTTTTTTGGCCGGCATGGTCCCAGCCTCCTCGCTGGCGCCGGCTGGGCAACATGCTTCGGCATGGCGAATGGGACggccGCTCTAGA***gctagc***

ssODN_e3_91: (5´-CTTCAAGCGGTCGAGGCCACAGCAAGGGTGTCGTGGTGTTCACCATTGATAAGTGACTAGGTAACTGAGTAGCACGGGGATTCTGGCTGGTACACAGTGTGCCAAGATTCCCTGACCC-3´)

ssODN_e3_46-91: (5´-AAAATTGCCTACAACGACGACCATCCACATGGACACGAGGATAAGTCTTCTAAGTGACTAGGTAACTGAGTAGCACGGGGATTCTGGCTGGTACACAGTGTGCCAAGATTCCCTGACCC-3´)

Key: Pink: guide RNA target sequence; Yellow: reverse complement of first 6 nucleotides of target; Blue: Hammerhead ribozyme; Teal: Hepatitis Delta virus ribozyme; Lower case: restriction endonuclease sites
